# The Feasibility of Supporting Caregivers of Young Children With Disruptive Behaviors Through Nurse-Delivered Phone Coaching: Quality Improvement Study

**DOI:** 10.2196/65244

**Published:** 2026-03-27

**Authors:** Hannah Mulholland, Jasmine Berry, Tammy Schmit, Barbara McIlrath, Jocelyn Lebow

**Affiliations:** 1 Department of Social Work, Mayo Clinic, Rochester, MN, United States; 2 Department of Psychiatry and Psychology, Mayo Clinic, 200 First Street SW, Rochester, MN, 55905, United States, 1 5072667808; 3 Department of Nursing, Mayo Clinic, Rochester, MN, United States

**Keywords:** nursing, telephone, parenting, pediatrics, toddler, health services accessibility, disruptive behavior, parents, quality improvement, coaching

## Abstract

**Background:**

Although childhood behavior problems are common, and strong evidence-based interventions exist to address these challenges, many families struggle to access care and remain in treatment long enough to see results. The Support and Advocacy through Providing Parents Helpful Interventions, Resources, and Education (SAPPHIRE) program was developed to address barriers to accessing care for disruptive behaviors in young children.

**Objective:**

This quality improvement program assessed the feasibility of SAPPHIRE, a primary care–based intervention delivered via telephone by trained nurses to caregivers of young children (n=36, ages 1‐6 y) who exhibit disruptive behaviors.

**Methods:**

The feasibility and acceptability of the SAPPHIRE program were assessed during a 3-month quality improvement study.

**Results:**

Of 36 participants, 25 (69%) completed the SAPPHIRE program. Over the course of 3 months, the number of nurse calls with completers ranged from 1 to 15, with a mean of 5.3 (SD 3.4) calls. Overall, nurses spent an average of 120.9 (SD 99.2, range 15‐380) minutes on the phone with each caregiver across the 3-month pilot period. Caregivers and nurses rated the program as acceptable across all metrics. For nurses, strengths of SAPPHIRE included the continuity of care with one family, while barriers included time constraints. Comparison of preintervention and postintervention caregiver ratings on measures of disruptive behaviors showed a moderate to negligible effect on reported behavior problems depending on the age of the child (children <4 y: *d*=0.55 and children 4‐6 y: *d*=0.18). Caregiver-rated parenting self-competence increased over the course of the SAPPHIRE intervention, approaching a large effect (*d*=0.75).

**Conclusions:**

Findings suggest that SAPPHIRE is a feasible and acceptable treatment for caregivers of young children with disruptive behaviors and shows promise for increasing parenting self-competence, which is a hypothesized moderator of future behavior problems. These preliminary data support the need for more rigorous empirical evaluation of the SAPPHIRE program.

## Introduction

Many young children struggle with disruptive behaviors (eg, physical and/or verbal aggression, defiance, etc) that impact their daily functioning at home and in school or daycare settings [1,2]. Prevalence rates of full-threshold disruptive behavior disorders in young children vary, but a recent meta-analysis estimates that as many as 4.9% of children between the ages of 1 and 7 years may meet the criteria for oppositional defiant disorder [2]. Although a spectrum of disruptive behaviors is considered normative in the developmental trajectory, children exhibiting these patterns of behavior—and their caregivers—can still benefit from interventions aimed at both decreasing disruptive behaviors and increasing family and caregiver functioning [3,4].

Even more, early childhood interventions targeting disruptive behaviors have been shown to decrease symptoms of early behavioral issues, bolster caregiver resiliency, and decrease the likelihood of later behavioral health diagnoses for their children [5,6]. Increasing accessibility and utilization of behavioral health treatment is particularly important, as studies support the efficacy of early childhood intervention in preventing later behavioral problems, with data suggesting that early emerging problem behavior in young children is malleable through family and caregiver-based interventions [7-9]. Data show that caregivers want opportunities to learn why their children are exhibiting disruptive behaviors and to develop skills to effectively respond to these patterns [10].

There are numerous effective interventions that target disruptive behavior in younger children; however, engagement in treatment may be limited due to a shortage of behavioral health providers trained in these modalities, as well as other accessibility challenges such as finding childcare, transportation, and cost [5,11-13]. Gold-standard interventions, such as Parent-Child Interaction Therapy [7], or Parent Management Training [14] are time- and resource-intensive, and mental health services are not always readily available in easy-to-access healthcare settings [11-13]. These interventions are thus characterized by access disparities, particularly across race and ethnicity [11,13]. Well-established interventions, such as Applied Behavioral Analysis, which are often offered in the home for families of children with behavioral concerns secondary to diagnoses including autism spectrum disorder, address some of these access issues by offering intervention in the family’s home environment [15]. These interventions, however, are costly and similarly difficult to access, which is why they are typically used for high rates of symptom severity [15]. Studies have shown significant rates of caregivers of children with disruptive behavior problems being lost to care when referred to behavioral health providers, both in community and primary care settings [13,16]. Accordingly, there is a substantial need for innovative care models that address barriers to access and engagement in treatment aimed at early intervention for young children with disruptive behavior problems.

One potential solution to improve access disparities is to integrate pediatric behavioral health services into the primary care setting [17]. Most youth have access to and visit primary care settings at least annually, so integrating behavioral health services into these clinics has the potential to reach a broad range of patients early in their symptom trajectory [18]. This approach has particular promise for addressing concerns about disruptive behavior, as studies suggest that a first step for many caregivers of children with disruptive behavior is to consult with their primary care provider [19]. As such, positioning low-impact interventions for disruptive behaviors at the point of primary care may increase families’ ability to follow through on receiving care [17,18,20-23].

Primary care–based behavioral health interventions have thus far been shown to be effective in decreasing and/or preventing depression, anxiety, and behavior issues in children and adolescents [20]. However, these approaches are not without their limitations regarding accessibility [22,23]. For one, most established primary care–based interventions for behavior problems exclude children younger than 5 years [24], precluding the use of those models as an early intervention. In addition, most models rely heavily on face-to-face, in-person care and the availability of embedded behavioral health providers to deliver the intervention. There is a relative dearth of literature examining alternative methods of behavioral health integration in primary care, such as using providers who may have more availability and are already staples of the primary care setting, such as nurses, or using alternative modes of treatment delivery, such as phone or video encounters, which reduce the need for in-person visits.

To our knowledge, only one group has developed a program designed to address this gap: the Strongest Families Smart Website (SFSW). This program, which was developed in Canada and further evaluated in Finland, includes 11 weekly web-based modules and accompanying telephone calls delivered by trained coaches, including nurses and individuals with public health backgrounds [25]. SFSW was found to have good acceptability and feasibility for parents of 4-year-olds with disruptive behavioral problems in Finland [26], and it demonstrated a significant impact on externalizing and internalizing behaviors that persisted after a 2-year follow-up period [26,27]. The promising results of this program lend support to the potential of phone- and web-based delivery of behavioral management strategies and suggest that nurses can be trained to effectively deliver coaching to help supplement skills training in this area. It should be noted, though, that the SFSW program remained a relatively sizable time commitment (an average of 451 min on web-based content and 418 min on calls) [26] and required sufficient staffing to deliver the coaching calls, which averaged 38 minutes per call. This may not be feasible for all health systems and potentially might be too much of a commitment for use as an indicated prevention or early intervention program for families of younger children and/or children with subclinical disruptive behavior concerns.

To address some of these gaps, the Support and Advocacy through Providing Parents Helpful Interventions, Resources & Education (SAPPHIRE) was developed—a primary care–based intervention that is delivered via brief telephone sessions with trained nurses. The program is designed to be used as part of a stepped care model for caregivers of young children (1‐6 y) who have disruptive behaviors that may place them at risk for disruptive behavior disorders in the future. SAPPHIRE uses well-established, evidence-based principles from behavioral parent training [14] and was designed to increase accessibility and engagement through innovations such as the phone-based, nurse-delivered format.

The primary aim of this quality improvement study was to evaluate the feasibility and acceptability outcomes of the SAPPHIRE treatment, including participant retention, as well as nurse and caregiver satisfaction. A secondary aim of this study was to examine the impact that the SAPPHIRE intervention had on parenting self-efficacy over the 3-month intervention period, as parenting self-efficacy has precedent as serving as a proximal risk factor or moderator for the severity of behavioral problems in young children [28]. It was hypothesized that there would be minimal immediate changes to child behavior during the 3-month treatment period due to SAPPHIRE being a brief, pilot intervention; however, improvement in parenting self-efficacy was expected.

## Methods

### Ethical Considerations

We assessed the feasibility and acceptability of SAPPHIRE. The Mayo Clinic Institutional Review Board reviewed this study and determined it should be designated as quality improvement and did not require further approval from the institutional review board or other ethics board. As this study was designated as quality improvement, no informed consent or assent was obtained; however, institutional guidelines for human subjects protection for quality improvement projects were followed at all times. No compensation was provided to participants.

### Sample, Setting, and Recruitment

Participants were recruited through referrals from pediatric and family medicine providers, including physicians, nurse practitioners, and physician assistants, within the participating primary care practice. All referring providers practiced in the same primary care practice associated with a tertiary medical center located in a small city in the Midwest United States. Eligible participants were parents or guardians of children aged 1 to 6 years who had concerns about their child’s behavior. Families already receiving treatment for disruptive behavior problems via a specialty service (eg, psychology, occupational therapy, sleep medicine, etc) were excluded. Aside from these exclusion criteria, providers offered the SAPPHIRE program to all eligible families presenting for a primary care visit between August 1, 2018, and July 31, 2021, with expressed concerns about their child’s behavior.

Initial evaluations were completed in-person or, after the onset of the COVID-19 pandemic, when the clinic gained the capacity for video visits, via video by a licensed clinical social worker embedded in the primary care practice. During this evaluation, the social worker completed a diagnostic assessment and provided psychoeducation on behavioral parent training principles [14]. The social worker was responsible for assessing the severity of disruptive behaviors, determining if there was an immediate need for a higher level of care, and evaluating the family’s ability and interest in participating in SAPPHIRE. The SAPPHIRE nurse then followed up with an initial phone call within approximately one week. Enrollment was contingent upon nurse availability. Given the high rates of staffing shortages due to both situational circumstances (eg, medical and parental leaves) and the impact of the COVID-19 pandemic, families were enrolled on a rolling basis, based on staff availability. Nursing staff typically followed 1 family at a time, with a maximum caseload of 2 concurrent families.

### Intervention

SAPPHIRE is a phone-based program in which pediatric primary care nurses provide support and coaching for families implementing behavioral parent training-based strategies. SAPPHIRE was developed in 2018, before the primary care clinic in question had the capacity to deliver video-based care. As such, the telephone was selected as the delivery method to be more convenient for families, as opposed to an in-person visit, which was the only other modality available at that time. The intervention itself was developed using evidence-based parenting principles [14], by board-certified child and adolescent psychologists and licensed clinical social workers with expertise in behavioral interventions for children. Feedback from experts in the fields of child psychology and primary care nursing was obtained and incorporated into the development of the program. During the 3-month intervention period, phone calls took place weekly or biweekly, based on caregiver schedules and nursing clinical judgment. Calls were supplemented by portal messages sent by nurses to summarize the content of each call, as well as to check in with the family during any gaps in contact. Caregivers could also reach out to their nurse-provider via portal or phone between scheduled sessions as needed.

At their intake appointment, families established 2 to 3 behavioral goals for their SAPPHIRE participation. These goals were based on evidence-based parenting principles, including increasing praise and special time, setting clear and consistent expectations, using selective attention, and implementing reinforcement schedules [29]. At the start of each nurse call, these treatment goals were reviewed, progress was measured, and barriers to success were problem-solved. If these initial goals were met, new goals were established with the SAPPHIRE nurse, with as-needed behind-the-scenes consultation with the social worker. Families did not meet with the social worker again during the SAPPHIRE program. When applicable, nurses sent patient education materials or community resources to caregivers following the calls. Nurses also provided support for caregivers by using strategies such as normalizing developmentally appropriate child behaviors and caregivers’ emotional responses to these behaviors, as well as providing education about typical childhood development.

### Training and Supervision

Six registered nurses with experience working in a pediatric primary care setting (experience ranged from 10 to 30 y) delivered the SAPPHIRE intervention. One nurse had experience working on a child and adolescent psychiatric inpatient unit for 3 years. The other nurses did not have a background in behavioral health. Training was conducted by a board-certified child and adolescent psychologist and a licensed clinical social worker with a specialty in child behavior problems. Training included: (1) readings on behavioral parent training and behavior change principles; (2) participation in 2-hour-long interactive sessions on behavioral parent training strategies and strategies for supporting caregivers, including active listening, normalizing behaviors, promoting self-care, and motivational interviewing; and (3) shadowing social workers for a minimum of 2 hours to observe behavioral parent training therapy sessions.

In addition, nurses participated in monthly hour-long group case consultations with a clinical psychologist and/or a licensed clinical social worker. Topics included case consultation, assessment of treatment adherence and fidelity, and review of SAPPHIRE skills. During this time, nurses reviewed cases and worked collaboratively with the psychologist and social worker to evaluate patients’ progress and re-evaluate the appropriateness of treatment goals and the SAPPHIRE program as a whole for the patients’ current symptoms. Additionally, the psychologist and social worker were available for as-needed individual case consultations between meetings.

### Measures

At baseline and at the end of treatment, caregivers completed one of two questionnaires assessing their concerns about their child. For older children (ages 4‐6 y), caregivers completed the Pediatric Symptom Checklist-17 (PSC-17) [30], a 17-item caregiver-report measure used to assess emotional and behavioral problems in children aged 4 years and older. The PSC-17 has been well-validated and contains 3 subscales—attention, internalizing, and externalizing—as well as a total score [31]. Caregivers of children younger than 4 years completed the Preschool Pediatric Symptom Checklist (PPSC) [32], an 18-item emotional and behavioral screening tool used for the early detection of emotional and behavioral problems in infants and preschoolers. This tool is widely used in primary care settings and contains 4 subscales: externalizing, internalizing, attention problems, and parenting challenges [32]. Additionally, at baseline and at the end of treatment, caregivers completed the Parenting Sense of Competence Scale [33], a 17-item measure that assesses self-efficacy in parenting.

### Data Collection

** **Caregivers completed all measures on paper. Families who were scheduled to be on-site for another medical appointment completed the surveys at that time. No families made a separate trip to the clinic to complete the measures. For families who were not planning to be on-site, paper measures were either mailed to them along with a self-addressed stamped envelope or sent as an attachment in their patient portal. Finally, an option to complete the surveys over the phone with a nurse was also offered. If caregivers selected this option, in order to minimize response bias, the nurse who assisted them with their forms was not the same nurse who delivered their SAPPHIRE intervention. Nurses worked with caregivers to determine which method of survey delivery was most convenient for the family.

### Data Analysis

Descriptive statistics were calculated to examine retention, patient or family characteristics, and treatment characteristics. In addition, Cohen *d* calculation for *t* tests was used to obtain the effect sizes of significant differences identified and was interpreted according to the following guidelines: 0.02=small effect, 0.05=moderate effect, 0.8=large effect [34].

## Results

### Participant Characteristics

In total, parents or guardians of 36 children (mean age=4.32, range: 1.42-6.92 y) were enrolled and participated in at least 1 nurse call as part of SAPPHIRE. The sample included 35 (97%) biological mothers and 1 (3%) biological father out of 36 caregivers. Children included 22 (61%) male-identifying children and 14 (39%) female-identifying children out of 35 children. The vast majority were White (34/36, 94%). Most had private insurance (25/36, 69%), followed by Medicaid (9/36, 25%) and Tricare (2/36, 6%). Children were given a range of *International Classification of Diseases, Tenth Revision* diagnoses, including problem behavioral child (18/36, 50%), any adjustment disorder (modifiers included with disturbed conduct, with mixed emotion and conduct, and undefined) (6/26, 17%), attention deficit/hyperactivity disorder or deficit of attention or concentration (3/36, 8%), developmental delay—speech (2/36, 6%), anxiety (1/36, 3%), and disruptive behavior disorder (1/36, 3%). Five out of 36 (14%) patients did not receive a diagnosis. See Table 1 for participant characteristics.

At baseline, 6 caregivers completed the PPSC with a mean score of 13.67 (SD 3.8). Scoring conventions suggest that scores higher than 9 on the PPSC indicate a child is “at risk” and warrant further intervention. Seventeen caregivers completed the PSC-17 for their children, with a mean global score at baseline of 12.94 (SD 5.8), which was slightly below the clinical cutoff of 15. Finally, 24 caregivers rated their parenting self-competence on the Parenting Sense of Competence Scale, with a mean baseline score of 68 (SD 9.6).

**Table 1.** Demographic and clinical characteristics of patients aged 1‐6 years old with behavioral concerns enrolled in the Support and Advocacy through Providing Parents Helpful Interventions, Resources, and Education (SAPPHIRE) program (N=36).

Characteristics of patients	Values
Age at baseline (y), mean (SD)	4.32 (1.55)
Race, n (%)	
White	34 (94)
Asian	2 (6)
Gender, n (%)	
Male	22 (61)
Female	14 (39)
Insurance, n (%)	
Private	25 (69)
Medicaid	9 (25)
Miscellaneous government	2 (6)
Primary diagnosis, n (%)	
Problem behavioral child	18 (50)
Adjustment disorder (with disturbed contact, with mixed emotion and conduct, undefined)	6 (17)
None	5 (14)
ADHD or deficit of attention or concentration	3 (8)
Developmental delay—speech	2 (6)
Anxiety	1 (3)
Disruptive behavior disorder	1 (3)

### Feasibility

Twenty-five out of 36 (69%) families completed the SAPPHIRE pilot program. Treatment completers were defined as families who successfully finished 3 months of the SAPPHIRE intervention or achieved all treatment goals before the 3-month period. Drop-outs were families who did not return for scheduled nurse calls and were lost to follow-up before achieving all treatment goals. Four out of 36 (11%) families were referred to a higher level of care (eg, Parent-Child Interaction Therapy or another more intensive behavioral therapy) before completing the program, and 7 out of 36 (19%) families dropped out without completing treatment after an average of 2.7 nurse calls, with a mode of 1 nurse call. One outlier family received 10 nurse calls before dropping out. Once they were removed from the analyses, the average number of nurse calls for dropouts was 1.5.

Over the course of 3 months, the number of nurse calls with completers ranged from 1 to 15, with a mean of 5.3 (SD 3.4) calls. In total, caregivers from this group had an average of 1.4 (SD 1.7) no-shows for scheduled calls. The mean number of portal messages sent was 2.2 (SD 2.9; range: 0‐14). Overall, nurses spent an average of 120.9 (SD 99.2, range: 15‐380) minutes on the phone with each caregiver across the 3-month pilot period. Table 2 describes SAPPHIRE usage data.

**Table 2.** Usage data from families enrolled in the Support and Advocacy through Providing Parents Helpful Interventions, Resources, and Education (SAPPHIRE) program (N=36).

	Mean (SD)	Range
Number of sessions	4.4 (3.4)	1‐15
Number of portal messages	2.2 (2.9)	0‐14
Number of no-shows	1.4 (1.7)	0‐8
Total time in intervention (min)	120.9 (99.2)	15‐380

### Acceptability

Caregivers rated the amount of time they spent participating in SAPPHIRE as “just right” and felt the phone-based format was “very convenient” (M=4.78/5). Overall, they thought the program was “very helpful” (M=4.61/5) and rated it as “very good” (M=4.78/5). Caregivers reported that, if they were to go back and do things over, they would be “very likely” (M=3.22/4) to participate in the program again and that they would “absolutely” recommend the program to another family of a young child with behavior concerns (M=3.5/4). See Table 3 for caregiver acceptability data. 

**Table 3.** Caregiver feedback on the acceptability of the Support and Advocacy through Providing Parents Helpful Interventions, Resources, and Education (SAPPHIRE) program.

Questions	Mean (range)
How serious do you currently consider your child’s behavior problem (0=not at all; 10=very serious, inpatient treatment necessary)?	3.5 (0‐8)
How strongly did you influence your child’s behavior during your time in the SAPPHIRE program (1=not at all; 5=very strong)?	3.5 (1-5)
How confident do you feel that you will be able to manage your child’s behavior in the future (1=not at all confident; 5=very confident)?	3.72 (2-5)
How time-consuming did you find participating in SAPPHIRE (0=far too time-consuming; 3=wish there was more time)?	2 (1-3)
How convenient was the phone-based care coordination (0=inadequate; 5=very)?	4.78 (4-5)
How helpful was SAPPHIRE (0=very unhelpful; 5=very helpful)?	4.61 (3-5)
How would you rate the program as a whole (0=inadequate; 5=very good)?	4.78 (4-5)
If you were to go back in time, would you participate in this program again (0=no way; 4=absolutely)?	3.2 (0‐4)
Would you recommend the program to another family whose young child has issues with behavior (0=no way; 4=absolutely)?	3.5 (2-4)

Caregivers also provided qualitative feedback on program acceptability. Caregivers felt that both the concrete suggestions for implementing and maintaining behavior plans, as well as the personalized support and reassurance, were the most helpful aspects of the program. Caregivers also commented on how the regular follow-up and accountability were very useful and how much they appreciated the continuity of care in meeting with the same nurse for every call.

The majority of nurses reported they were “satisfied” with being a part of the program (M=4/5), and half felt it increased their job satisfaction. The other half reported that their participation in the program had no impact on their job satisfaction. Nurses largely felt they had adequate training to deliver the interventions (M=2.5/3), and all felt they had adequate support through as-needed consultation with specialists and case consultation meetings. When asked what they liked most about the program, nurses reported finding it rewarding to be able to follow families over time and having continuity of care. They reported feeling like they were helping families and making a difference. One nurse stated that she particularly enjoyed “being able to help parents with their concerns. When I was able to get a hold of parents, providing some specific things to try and providing reassurance was rewarding.”

Nurses also described challenges with the program, including finding time to make the calls and focus on their cases amidst their other job responsibilities. For example, one nurse noted that she felt there was “not enough time to focus on multiple (SAPPHIRE) patients due to other job responsibilities.” They provided feedback that some of the processes around enrollment and data collection were time-consuming, and they discussed the difficulties of playing “phone tag” when families were unavailable during scheduled calls. See Table 4 for nurse acceptability data.

**Table 4.** Nurse feedback on the acceptability of the Support and Advocacy through Providing Parents Helpful Interventions, Resources, and Education (SAPPHIRE) program.

Questions	Mean (range)
Please rate your satisfaction in being a part of the SAPPHIRE Program. (1=very unsatisfied; 5=very satisfied).	4 (3-5)
How did participating in the SAPPHIRE Pilot impact your job satisfaction? (1=decrease job satisfaction; 3=increase job satisfaction)?	2.5 (2-3)
Did you feel you had adequate training in SAPPHIRE strategies prior to seeing patients? (1=no; 3=yes)?	2.5 (1-3)
Did you feel you had adequate support from specialists (eg, social worker and/or psychologist) for as-needed case consultation during the intervention? (1=no; 3=yes)?	3 (3-3)
Were the amount of case consultation meetings (1=too few, 2=just right; 3=too many)?	2 (2-2)
What did you like most about participating in the SAPPHIRE pilot?	I get to use my previous psychology experience and work toward my license. I enjoy getting to know these families and helping them and their children succeed. Relating to parents, as I have children the same age. Continuity of care. Being able to help parents with their concerns. When I was able to reach parents, providing some specific things to try and providing reassurance was rewarding. Reinforcing the tools that social work had discussed with parents in the office. Felt like I was helping parents and patients and making a difference.
What did you like least about participating in the SAPPHIRE pilot?	Not enough time to focus on multiple patients due to other job responsibilities. I would like the referral process to be better streamlined. Times when there was no warm handoff; inconsistency with enrolling patients (the email system did not work) Not my area of clinical knowledge, and I did not like the work When unable to reach parents, even when a date and time were set for a phone visit. Finding time to do it and playing “phone tag” with parents. Finding time to complete phone calls and calling families to complete the long surveys.

### Preliminary Outcomes

At the end of treatment, caregivers stated that they felt they had a “quite strong” to “strong” influence on their child’s behavior during the course of the program (M=3.5/5) and felt “confident” that they would be able to manage their child’s behavior in the future (M=3.72/5). They rated their child’s current behavior problem as “slightly serious (no intervention necessary)” (M=3.5/10).

 Comparison of preintervention and postintervention caregiver ratings on both the PPSC and PSC-17 showed decreases in reported behavior problems. The effect size for the decrease in behavior problems among younger patients who were rated with the PPSC (n=8) exceeded Cohen’s convention for a moderate effect (*d*=0.55). For the sample of older children (n=7), whose behavior was rated using the PSC-17, there was a negligible effect (*d*=0.18). Similarly, caregiver-rated parenting self-competence increased over the course of the SAPPHIRE intervention. The effect size for the increase in parenting self-efficacy approached a large effect (*d*=0.75). See Table 5 for preliminary outcomes.

**Table 5.** Preliminary outcome data for patients completing the Support and Advocacy through Providing Parents Helpful Interventions, Resources, and Education (SAPPHIRE) program.

Measures	Baseline		End of treatment		Effect size
	Mean (SD)	n	Mean (SD)	n	
Preschool Pediatric Symptom Checklist (PPSC)	13.67 (3.8)	6	10.38 (7.5)	8	0.55
Pediatric Symptom Checklist-17 (PSC-17) total score	12.94 (5.8)	17	11.86 (6.1)	7	0.18
Parenting Sense of Competence Scale (PSOC)	68 (9.6)	24	75.5 (10.4)	16	0.75

## Discussion

### Principal Findings

 Findings suggest that the SAPPHIRE program is a feasible intervention for caregivers of young children with disruptive behaviors. Overall, 69% (25/36) of caregivers completed the program, which compares favorably to the estimated 49% reported retention rate in larger studies of conventional disruptive behavior interventions [11] and is slightly less favorable than the 75% to 77.6% retention rate of the Canadian and Finnish implementations of the web- and phone-based SFSW program [25,26]. It is unclear whether differences in retention were due to characteristics of the program, cultural differences, or possibly differences in assessment periods (eg, part of the SAPPHIRE quality improvement study overlapped with the COVID-19 pandemic). Future evaluation of this is needed to clarify and address barriers to retention in the SAPPHIRE program, as well as to establish the effectiveness of the intervention in a larger sample.

SAPPHIRE required relatively few resources compared to other disruptive behavior programs, as nurses spent an average total of slightly more than 2 hours on calls with a family. This compared favorably with the approximately 7 hours of phone coaching included in the SFSW program [25,26], though it may have implications for retention that should be explored. Results also suggest that both nurses and participating caregivers found SAPPHIRE acceptable. Caregiver feedback reflects that aspects of the program that were particularly well-received included the regular follow-up, continuity of care, and focus on concrete interventions. Nursing staff also reported good satisfaction with the program, though time for implementation was cited as one of the barriers that might impact future dissemination in primary care settings.

 The effect of SAPPHIRE on disruptive behavior was moderate for younger children and negligible, approaching small, for older children. These differences in effect sizes should be interpreted with caution, given the fact that younger and older children were evaluated using distinct measures with different scoring conventions and clinical cutoffs. However, these findings align with the literature, which suggests that younger children are typically more responsive to behavioral interventions [35]. Given that the program was implemented over the course of 3 months, which is likely too brief a time period to have a substantial impact on behavioral patterns, we would not necessarily expect to see large effects on behavior. This may explain why younger children whose parents completed the PPSC screener continued to score slightly above the “at-risk” cutoff of 9 at the end of treatment and why the intervention had a negligible-sized effect on the PSC-17 total score. The 3-month study period was chosen based on quality improvement pilot study parameters rather than clinical considerations. Caregivers expressed some dissatisfaction with the brevity of the program, though feedback also suggests that weekly phone calls may not always be necessary and that it may be possible to decrease the frequency of contact as the program progresses. Future iterations of SAPPHIRE would likely benefit from offering families the option to enroll on a flexible call schedule for longer periods of time, as needed.

It is also possible that effects on behavior were not observed in older children whose symptoms were measured by the PSC-17 because baseline scores for this group were only somewhat elevated and did not exceed clinical cutoffs. As SAPPHIRE was developed as an early intervention or indicated prevention option for children who have not yet developed significant behavior problems that warrant a higher level of intervention, it is important to note that even small decreases in reported behavioral concerns are potentially clinically significant. These results suggest that there is merit in further evaluating SAPPHIRE’s effectiveness and that a longer follow-up assessment period is needed to determine whether the intervention prevents the onset of more serious disruptive behaviors in the future. Though we might not expect major changes in child behaviors after the 3-month SAPPHIRE pilot, we would expect to see larger effects on proximal risk factors, such as caregiver self-competence. As such, the fact that the intervention approached a large effect on this variable (*d*=0.75) is promising, as are caregiver self-reports of increased confidence in their ability to positively influence their child’s behavior moving forward.

 Overall, findings support the feasibility and acceptability of the SAPPHIRE program, and preliminary outcome measurements suggest it may positively impact parenting self-competence. It is possible that, as caregivers attested, the accountability and regular check-ins made possible by the phone-based format of SAPPHIRE helped caregivers apply behavioral parenting strategies consistently and allowed for real-time adjustments to behavior plans and treatment goals, which increased the likelihood of caregiver follow-through. Patient retention and compliance are major challenges of established, highly effective, evidence-based behavioral interventions. The SAPPHIRE format of nurse-led, phone-based coaching calls may potentially avoid some of the common barriers to accessing and remaining in traditional behavior therapies, including mitigating the difficulties inherent in coordinating in-person sessions while simultaneously trying to parent a young child with challenging behaviors.

There were important lessons learned that should be taken into account when considering the feasibility of this program moving forward. Notably, the field of nursing continues to be characterized by high demand, high turnover, and inadequate staffing models. We found this to be true with SAPPHIRE, as we were often delayed in enrolling patients due to nursing staffing shortages. At several points in the program, new cohorts of nurses had to be trained to provide the care. Though training was efficient and SAPPHIRE itself took a relatively brief amount of time, it still is an additional demand on the already overburdened primary care nurse’s schedule. Nurses noted that the time burden of the intervention was still a limiting factor. As such, future iterations of the intervention would benefit from a stakeholder-engaged redesign, in which primary care nursing, nurse administrators, and caregivers are involved in brainstorming ways to further streamline and minimize burdens inherent in finding time for phone calls, playing phone tag, etc.

### Limitations

This quality improvement study has several limitations, most notably that no conclusions about program effectiveness can be drawn from this study design, given the small sample. Interpretation of these results must also account for the homogeneous sample, particularly with regard to race and ethnicity, and the fact that the feasibility and acceptability of this study are limited by the brief, 3-month intervention period. In addition, the study time period was characterized by nurse staffing shortages which, while typical for a primary care setting, particularly during the time period that included the onset of the COVID-19 pandemic, prolonged the study enrollment period. This may have led to some time-modified confounding factors that impacted our findings. Patient characteristics and caregiver availability to participate in SAPPHIRE in the last year of this study may have been impacted by the dramatic demands placed on families by the COVID-19 pandemic. Because of this, all findings should be interpreted as preliminary, and the need for additional systematic evaluation of this program cannot be overstated. Even with limitations, however, this study effectively highlights the potential benefit of nurse-led interventions in the primary care setting.

### Conclusion

In sum, due to robust support for behavioral parenting programs in improving childhood behavior problems, and despite considerable issues with access and retention for these interventions, the SAPPHIRE treatment model has potential for filling a much-needed treatment-access gap as an early intervention or indicated prevention program for first developing or subclinical behavior problems in young children. Our findings suggest that SAPPHIRE is feasible and acceptable for caregivers of young children and shows promise for increasing parenting self-competence and improving child behavior problems. Although these data are preliminary, they provide support for more rigorous empirical evaluation to examine whether SAPPHIRE is an effective treatment option for this population.

## Abbreviations

PPSC: Preschool Pediatric Symptom Checklist
PSC-17: Pediatric Symptom Checklist-17
SAPPHIRE: Support and Advocacy through Providing Parents Helpful Interventions, Resources, and Education
SFSW: Strongest Families Smart Website
